# Investigating the Correlation between Electrolyte Concentration and Electrochemical Properties of Ionogels

**DOI:** 10.3390/molecules28135192

**Published:** 2023-07-04

**Authors:** Ji Wei Suen, Naveen Kumar Elumalai, Sujan Debnath, Nabisab Mujawar Mubarak, Chye Ing Lim, Mohan Reddy Moola, Yee Seng Tan, Mohammad Khalid

**Affiliations:** 1 Department of Mechanical Engineering, Faculty of Engineering and Science, Curtin University, Miri 98009, Malaysiad.sujan@curtin.edu.my (S.D.);; 2 Energy and Resources Institute, Faculty of Science and Technology, Charles Darwin University, Darwin, NT 0909, Australia; 3 Petroleum and Chemical Engineering, Faculty of Engineering, Universiti Teknologi Brunei, Bandar Seri Begawan BE1410, Brunei; mubarak.yaseen@gmail.com; 4 Research Centre for Crystalline Materials, School of Medical and Life Sciences, Sunway University, Bandar Sunway, Petaling Jaya 47500, Malaysia; 5 Sunway Centre for Electrochemical Energy and Sustainable Technology (SCEEST), School of Engineering and Technology, Sunway University, Bandar Sunway, Petaling Jaya 47500, Malaysia; 6 Division of Research and Development, Lovely Professional University, Phagwara 144411, Punjab, India; 7 School of Applied and Life Sciences, Uttaranchal University, Dehradun 248007, Uttarakhand, India

**Keywords:** ionogel, ion pairs, specific capacitance, ionic conductivity

## Abstract

Ionogels are hybrid materials comprising an ionic liquid confined within a polymer matrix. They have garnered significant interest due to their unique properties, such as high ionic conductivity, mechanical stability, and wide electrochemical stability. These properties make ionogels suitable for various applications, including energy storage devices, sensors, and solar cells. However, optimizing the electrochemical performance of ionogels remains a challenge, as the relationship between specific capacitance, ionic conductivity, and electrolyte solution concentration is yet to be fully understood. In this study, we investigate the impact of electrolyte solution concentration on the electrochemical properties of ionogels to identify the correlation for enhanced performance. Our findings demonstrate a clear relationship between the specific capacitance and ionic conductivity of ionogels, which depends on the availability of mobile ions. The reduced number of ions at low electrolyte solution concentrations leads to decreased ionic conductivity and specific capacitance due to the scarcity of a double layer, constraining charge storage capacity. However, at a 31 vol% electrolyte solution concentration, an ample quantity of ions becomes accessible, resulting in increased ionic conductivity and specific capacitance, reaching maximum values of 58 ± 1.48 μS/cm and 45.74 F/g, respectively. Furthermore, the synthesized ionogel demonstrates a wide electrochemical stability of 3.5 V, enabling diverse practical applications. This study provides valuable insights into determining the optimal electrolyte solution concentration for enhancing ionogel electrochemical performance for energy applications. It highlights the impact of ion pairs and aggregates on ion mobility within ionogels, subsequently affecting their resultant electrochemical properties.

## 1. Introduction

Stretchable and flexible electronic devices are emerging as new types of intelligent devices, which have grown enormously due to their real-time feedback, flexibility, and portability. Current flexible wearable devices can transmit, analyze and monitor human physiological signals in real time, presenting significant advantages [1]. Traditional sensors made of semiconductors and metals have restricted their application in wearable electronic devices due to poor stretchability and flexibility [2]. The fundamental aspect of the sensor is the establishment of deformation-responsive and conductive materials. The common practice for producing such materials is by incorporating electroactive materials into a polymer matrix, such as silver nanoparticles, graphene and carbon nanotubes [3,4].

Nevertheless, the escalation of ionogels has acted as a new initiative for application in energy [5] and wearable electronic devices. Ionogels are gels materials, whereas ionic liquids are constrained in a 3D crosslinking network through different interactions, forming desirable structures [6]. Ionogels maintain the interesting properties of ionic liquids, including high ionic conductivity, [6] wide electrochemical stability, resistance to evaporation and being highly tunable [7]. Thus, combining outstanding properties has made ionogel an exciting candidate for application in flexible solar cells [5] and other electronic devices [8].

The properties mentioned above result from the presence of ionic liquid within ionogel. Confinement of ionic liquids within ionogel adversely affects the electrochemical properties of ionogel, especially its ionic conductivity. The decrease in ionic conductivity is due to adding polymer, which will increase viscosity, thus prohibiting ion transportation [9]. Numerous techniques have been explored to boost ionogel performance, and one such method involves the addition of lithium salt. Research indicates that incorporating lithium salt can strengthen the electrochemical stability of ionogels. In addition, the dissolved lithium salt facilitates the conduction mechanism by establishing pathways for anions and cations, thereby increasing ionic conductivity [10]. To date, lithium salts have been incorporated in the synthesis of ionogel. For instance, Guillemin et al. studied the impact of adding lithium salts on the performance of an ionogel-based micro-supercapacitor [11]. The addition of lithium salt to ionogel notably boosts the energy density and capacitance of micro-supercapacitors (2.96 μWh cm^−2^ to 10.22 μWh cm^−2^), validating their use in high-temperature applications. Cheng et al. examined the impact of different lithium salts (LiOTf, CF_3_LiO_3_S and LiTfSI, LiC_2_NO_4_F_6_S_2_) on ionogel thermal stability and electrochemical performance [12]. The addition of lithium salts initiated the formation of a porous cross-linked structure, which is beneficial for ion transportation; thus, both lithium salt-incorporated ionogels have achieved outstanding ionic conductivity (10^−3^ S cm^−1^). Ogawa et al. proposed radical polymerization of ionic liquid in the presence of lithium salt and active material without solvent [13].

It has been demonstrated that the incorporation of lithium salt in ionogel leads to promising electrochemical properties of the ionogel. However, limited study has been conducted on understanding the interaction between lithium salt and ionic liquid. The possible interactions within ionogel are vital as these interactions may influence the performance of the ionogel. Hence, this study aims to comprehend the interaction between lithium salt and ionic liquid and the possible effect on the electrochemical performance of ionogel. In this study, the ionogel is synthesized through a non-aqueous sol-gel route. The 1-ethyl-3-methylimidazolium bis (trifluoromethyl sulfonate) (EMIM TFSI) is an ionic liquid. Lithium trifluoromethyl sulfonate, LiTf acts as a lithium salt. In contrast, Tetraethyl orthosilicate (TEOS) and Poly(vinylidene fluoride)-co-hexafluoropropylene (PVDF-HFP) are used as silica precursor and polymer matrix, respectively. The interaction between LiTf and EMIM TFSI is investigated by manipulating the concentration of electrolyte solution in the ionogel, which is then analyzed with a series of material and electrochemical characterization studies.

## 2. Results and Discussion

**Figure 1:**
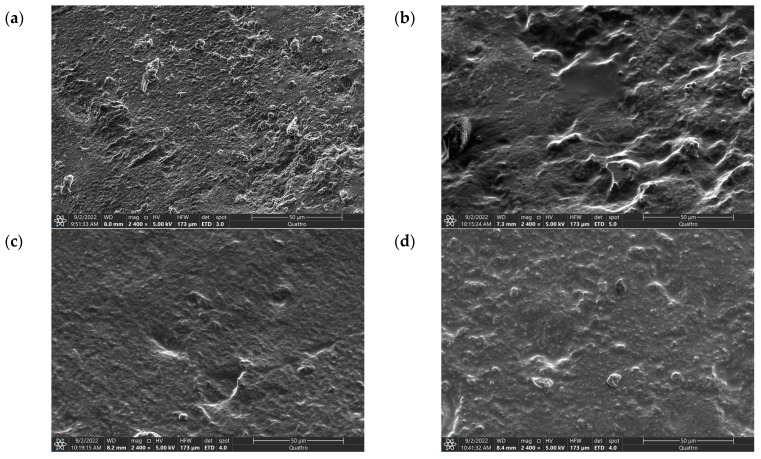
SEM Images of (**a**) IG-25, (**b**) IG-28, (**c**) IG-31, (**d**) IG-38.

Scanning electron micrographs were used to study the morphology of ionogels. The ionogel was characterized by scanning electron microscopy, and the images were captured at the same magnification for comparison. Figure 1 shows the SEM images of different concentrations of electrolyte solution.

The SEM image of IG-25 shows a rough and uneven morphology. Besides the rough surfaces, several protrusions can be observed from the morphology of IG-25, while these protrusions exhibit a rough surface. This observation suggests the dominating characteristic of PVDF-HFP at low concentrations of electrolyte solution. As for IG-28, fewer protrusions have been observed on the surface of the ionogel. Instead, the protrusions have a smoother surface. This phenomenon could be due to the electrolyte solution being entrapped within the ionogel system. As the concentration of electrolyte solution increases, this forms an interconnected film within the polymer matrix. The 3D interconnected polymer–electrolyte solution structure implies the complete confinement of the electrolyte solution within the polymer matrix [14]. At 31 vol% of electrolyte solution, the effect of the electrolyte solution is more pronounced, whereby a smoother surface is exhibited on the morphology of ionogel. The presence of a smooth surface is ascribed to the addition of an electrolyte solution that reduces the crystallinity of the ionogel [15]. Furthermore, the smooth surface morphology also suggests a homogenously dispersed network [16]. Thus, it can be anticipated that the ionic conductivity will increase in the initial increment of electrolyte solution due to reduced crystallinity and a homogeneously dispersed network that provides a pathway for ion transportation. However, small aggregates can be observed at a concentration higher than 31 vol%. The formation of aggregates could be due to ionic liquid aggregation, which impedes ionic conductivity. Nevertheless, this will be further investigated and evidenced through XRD and FTIR.

**Figure 2:**
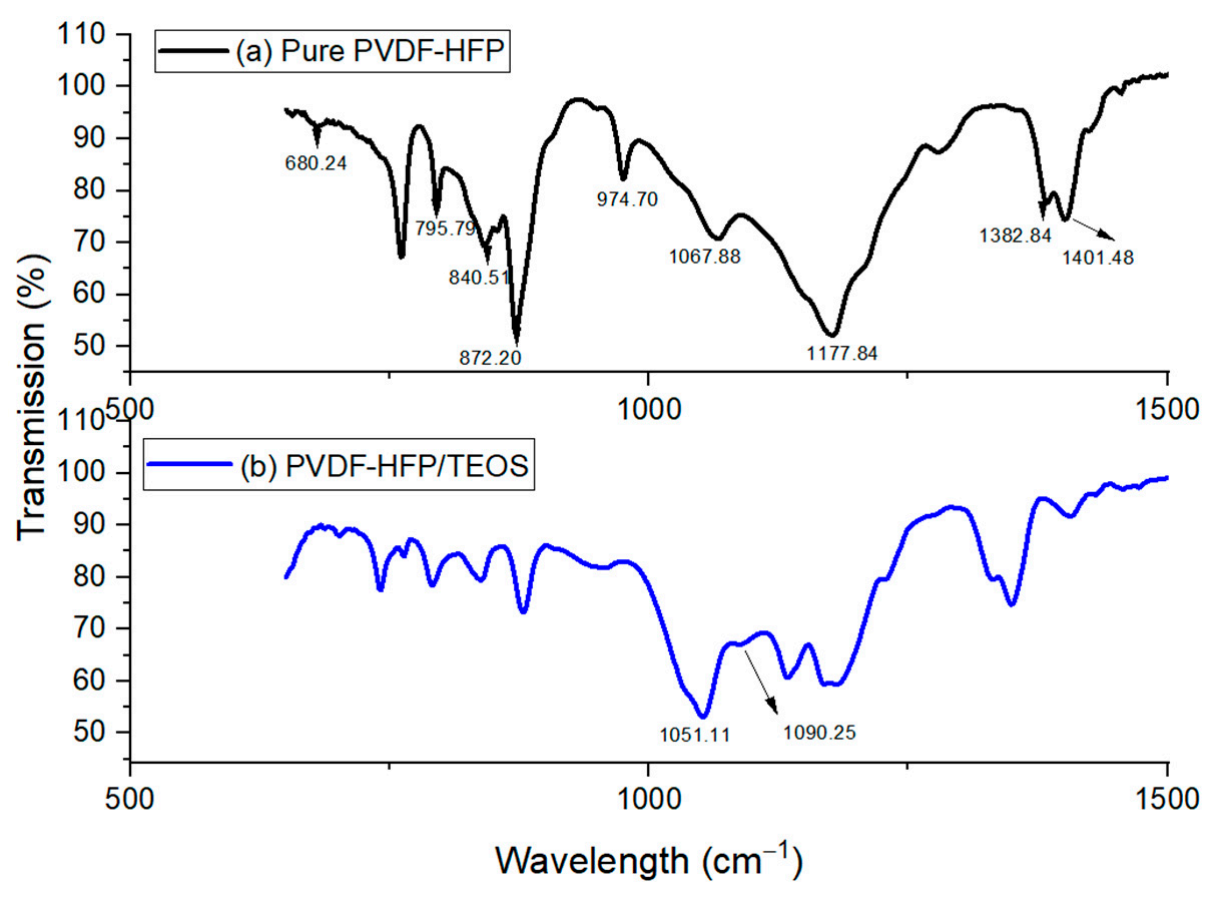
FTIR Spectra for (**a**) Pure PVDF-HFP (**b**) PVDF-HFP/TEOS.

Fourier transform infrared spectroscopy (FTIR) characterization is performed on the samples to study the structural changes of the ionogel, which can be correlated to the electro-mechanical performances. Figure 2 shows the FTIR spectra of pure PVDF-HFP.

PVDF-HFP is an interesting polymer that displays different polymorphs, such as the alpha (α) phase, beta (β) phase and gamma (γ) phase. The classification of phases is based on the chain conformation; for instance, the alpha phase is in the trans-gauche-trans-gauche’ conformation, while the beta phase has an all-trans planar zigzag conformation [17,18]. Efforts have been devoted to understanding the formation of varied phases, which is crucial in their wide extensive application. Meanwhile, this can be evidenced by the FTIR characterization. Bands were observed at 840.51 cm^−1^, 872.20 cm^−1^, 1067.88 cm^−1^, 1177.84 cm^−1^ and 1401.48 cm^−1^, respectively. These bands serve as an indication of the beta phase in PVDF-HFP [19]. Furthermore, the bands observed at 741.74 cm^−1^ and 840.51 cm^−1^ are accountable for the rocking vibration of CH_2_ [19]. Regarding vibration modes, the bands observed at 1401.48 cm^−1^ are due to the symmetric stretching vibrations of CH_2_. The IR bands confirm the mixed mode of combined CC and CF_2_ symmetric stretching observed at 872.20 cm^−1^ and 1067.88 cm^−1^ [19]. Apart from that, the characteristics of vibrational peaks observed at 1401.48 cm^−1^, 1177.84 cm^−1^ and 1067.88 cm^−1^ were due to the vibration of CF_2_ groups, swinging vibrations of CH_2_ and C-C-C bending vibration. In short, different bands are due to various vibrational modes and are also accountable for different phases, with bands observed at 1067.88 cm^−1^ non-polar crystalline α phase. Meanwhile, a weak band is observed at the peak of 840.51 cm^−1^, which indicates a mixed mode of beta phase (β) of the PVDF-HFP [20,21].

Figure 2b shows the spectrum for PVDF-HFP with TEOS. A small peak was observed in the spectrum after the addition of TEOS, at a wavelength of 1090.25 cm^−1^; this is due to asymmetrical vibration stretching of Si-O-Si [20,22]. The appearance of a new peak implies the formation of silica particles within the PVDF-HFP matrix. Concerning the peak shifting, the initial peaks at 1052.97 cm^−1^ have been found to shift to 1051.11 cm^−1^, indicating the interaction of Si from SiO_2_ with the F of the CF3 group from PVDF-HFP. Moreover, the presence of SiO_2_ is also verified by observing absorption bands 990 cm^−1^ to 1135 cm^−1^, where a weak band can be observed near 957.93 cm^−1^; this indicates the weak bending vibration Si-OH. In addition, Si-O-Si asymmetric stretching vibration can be reported in the spectrum range of 838.65–764.10 cm^−1^.

**Figure 3:**
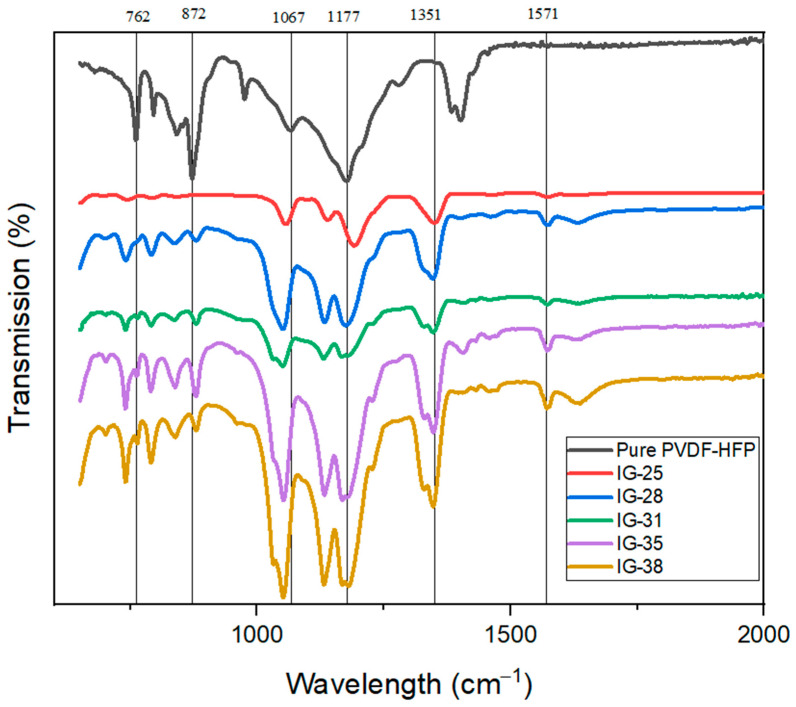
FTIR spectra for Pure PVDF-HFP, IG-25, IG-28, IG-31, IF-35, IG-38.

Figure 3 shows the FTIR spectra for pure PVDF-HFP and Ionogel with different concentrations of electrolyte solution. For the FTIR spectra for pure PVDF-HFP, two prominent peaks at 762.24 cm^−1^ and 872.19 cm^−1^ indicate the presence of the beta phase. However, after incorporating an electrolyte solution, both peaks exhibit a drastic reduction in intensity. The FTIR graphs show this peak’s intensity. Nevertheless, a slight shift is observed at peak 872.19 cm^−1^ after the incorporation of electrolyte solution where the peak is shifted to 879.65 cm^−1^. This phenomenon is consistent with previously reported findings, implying the presence and complexation of salt with the host polymer matrix. Furthermore, a peak at 1067.88 cm^−1^ was observable for pure PVDF-HFP, but this peak disappears for FTIR spectra of ionogel. Instead, for ionogel, with a new peak at 1054.83 cm^−1^, the presence of a new peak is also due to TFSI anion in the ES [23,24]. However, when the concentration of electrolyte solution reaches 31 vol%, a new shoulder peak (1028.75 cm^−1^) appears adjacent to the peak at 1054.83 cm^−1^. Nevertheless, peaks at band 1067.88 cm^−1^ and 1177.84 cm^−1^ remain in the FTIR spectra of the ionogel, showing good compatibility between an electrolyte solution and polymer matrix. The new peaks at 1347.43 cm^−1^ correspond to conformation, stretching and anti-stretching of the TFSI anion [25]. Moreover, a new peak is observed at 1571.09 cm^−1^ due to the stretching of EMIM^+^ cations, evidenced by the ionic liquid in ionogel. This peak becomes more dominant as the amount of the electrolyte solution increases [20,26,27].

**Figure 4:**
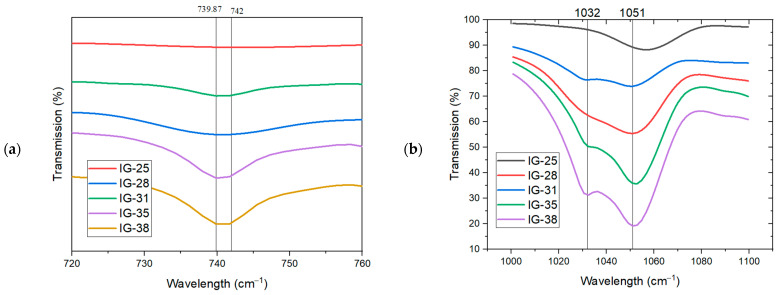
FTIR Spectra in the wavelength of (**a**) 700 cm^−1^ to 760 cm^−1^ and (**b**) 1000 cm^−1^ to 1100 cm^−1^.

To further understand the presence of free ions or the formation of ion pairs, the FTIR spectra in the wavelength of 700 cm^−1^ to 770 cm^−1^ and 1000 cm^−1^ to 1100 cm^−1^ were displayed in Figure 4a. From Figure 4a, the wavelength at ~740 cm^−1^ is used to comprehend the availability of TFSI anion in the ionogel. As the concentration of the electrolyte solution increases from 25 vol% to 33 vol%, the peak at 739.87 cm^−1^ accounts for the increased availability of free TFSI- anions. Meanwhile, a second peak appears at a wavelength of 742 cm^−1^ due to the formation of ion pairs or ion aggregation in the ionogel. This peak becomes more pronounced when the electrolyte solution concentration reaches 38 vol%. As the concentration of the electrolyte solution continues to increase, the intensity of this peak is also enhanced, suggesting more ion pairs/ion aggregates present in the ionogel at a high concentration of electrolyte solution.

From Figure 4b, the peak at 1032 cm^−1^ is ascribed to the free ions present in the ionogel [21,28]. At low concentrations of electrolyte solution (25 vol%), this peak is faint, suggesting a scarcity of free ions in the ionogel. Further increment in the concentration of electrolyte solution results in the intensity enhancement of this peak, implying the presence of more free ions. On the contrary, as the concentration increases, a new peak appears at 1054 cm^−1^, resulting from forming ion pairs/ion aggregates. The intensity of this new peak eventually surpasses the peak at 1032 cm^−1^, which infers that there are more ion pairs/ion aggregates than free ions present in this ionogel. Thus, FTIR spectra suggest that at lower concentrations of electrolyte solution (25 vol% to 31 vol%), more free ions are available in the ionogel system. Nevertheless, further increasing the concentration of electrolyte solution, ions tend to interact with each other, forming ion pairs, or ion aggregates, as evidenced in the peak intensity at 742 cm^−1^ and 1054 cm^−1^, respectively.

**Figure 5:**
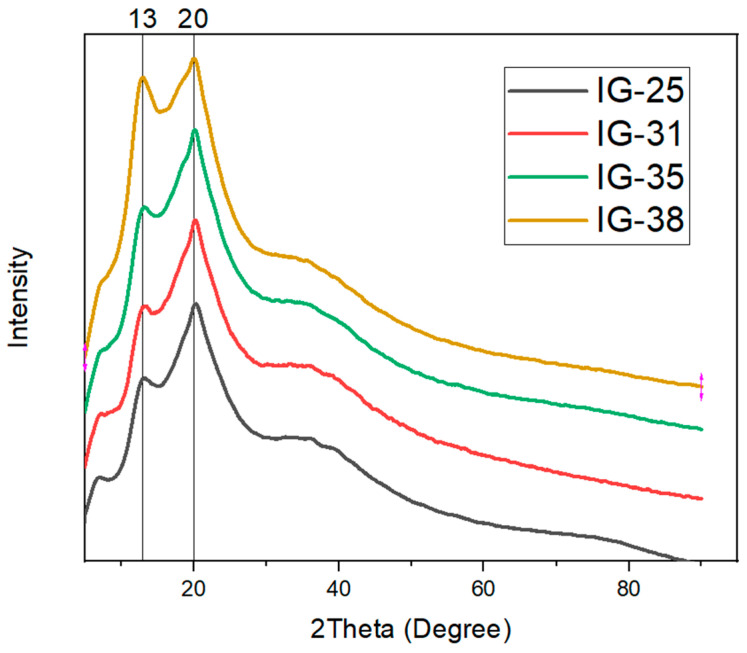
XRD Curve for Ionogel with x-vol% of electrolyte solution.

The X-ray diffraction characterization was performed to study the crystallinity of ionogel. The degree of crystallinity refers to the degree of the structural ordering within the ionogel. Figure 5 depicts the XRD curve for the ionogel.

Based on the XRD curves for all ionogels, two prominent peaks are observable at around 13° and 20°. In addition to the two distinct peaks, a broad hump is observed near 40°. The two prominent peaks are contributed by the α-phase crystals and β-phase crystals from PVDF-HFP, which demonstrate the semi-crystalline structure of PVDF-HFP [29]. However, the broad hump near 40° suggests the amorphous phase in the ionogel. As the concentration of electrolyte solution increases, one noticeable change is the enhancement of the broad peak near 40°. This phenomenon suggests that the increasing concentration of electrolyte solution enhances the amorphous phase of ionogel, which is favorable for the mobility of ions. This enhancement of the amorphous phase is ascribed to the Lewis acid-base reaction upon the addition of electrolyte solution, wherein the electrolyte solution acts as a Lewis acid. Simultaneously, the polymer matrix is considered Lewis’s base [30,31]. Besides the amorphous phase enhancement, the first peak (13°) has also increased. The intensity augmentation of the first peak is attributed to the high concentration of electrolyte solution; such a phenomenon evinces improved crystallinity [32]. Furthermore, the monophasic ionic liquid could also contribute to the observed phenomenon [33,34]. To illustrate, the degree of crystallinity is evaluated and tabulated in Table 1.

According to Table 1, the highest degree of crystallinity was shown by IG-25, followed by a drastic reduction when the concentration of electrolyte solution increased up to 31 vol%. This reveals that adding more electrolyte solution reduces the crystallinity of the ionogel, which is beneficial for ion transportation. However, beyond the concentration of 31 vol%, the degree of crystallinity continues to escalate and attain the highest degree of crystallinity (63.51%) at 38 vol%. Thus, adding electrolyte solution has enhanced the amorphous phase in the ionogel. At the same time, a further increase may result in increased crystallinity, which is undesired as this may lower the ionic conductivity.

**Figure 6:**
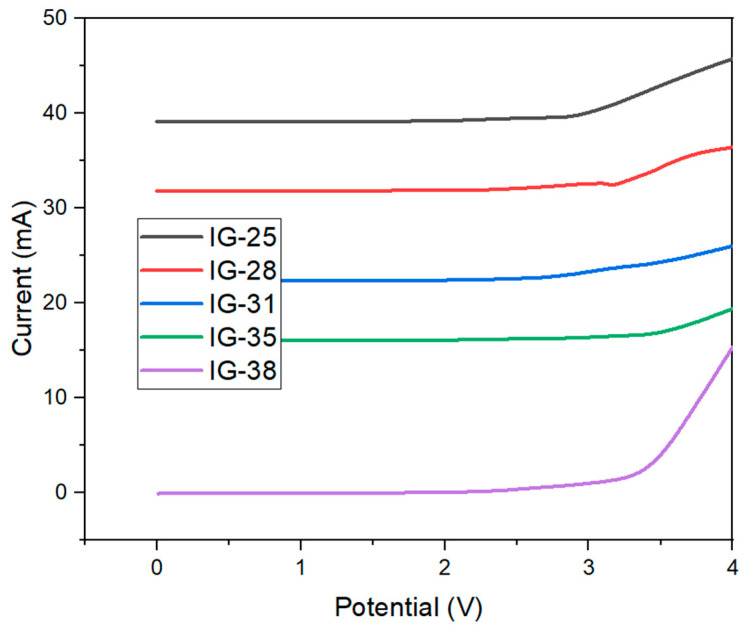
Linear Sweep Voltammetry Plot of Ionogel.

The electrochemical stability window (ESW) is an underlying consideration in evaluating the performance of ionogel as a solid electrolyte. Linear Sweep Voltammetry is a common technique to identify the electrochemical stability of window ionogel. Figure 6 shows the linear sweep voltammetry graph for all ionogels. The ionogel was tested in the voltage range of 0–5 V, as high voltage may lead to the decomposition of ionogel. All the ionogels showed a similar trend, whereby no current was captured when the voltage was applied, up until 3 V, where a drastic increase in the current is observed as the voltage is increased. The sudden spike in current is due to the decomposition of polymer electrolytes [35]. Similar LSV plots for ionogel have been reported [29,36,37,38]. The electrochemical stability window is identified by drawing a tangent on the uprise of the curve and intercepting the voltage axis [39].

The electrochemical stabilities are summarized in Table 2. From Table 2, it is observable that with the increase in the concentration of electrolytes solution, no substantial increment was observed in the electrochemical stability of ionogel. The results show that for electrolyte solutions in the range 25–38 vol%, the electrochemical stability is reported to be within the range of 3.00–3.50 V. To conclude, no significant current was recorded within 0–3.50 V, indicating that the synthesized ionogel has shown good electrochemical stability up to 3.50 V. The wide electrochemical stability window exhibited by ionogel has allowed it to be applied in photonic devices [40], common electrical double-layer capacitors and energy storage devices [41].

Moreover, an increasing trend in electrochemical stability can be observed as the concentration of electrolyte solution increases. The maximum electrochemical stability, 3.50 V, is achieved by IG-31, followed by a subsequent decreasing trend. The electrochemical stability of ionogel is influenced by the ionic liquid due to strong interactions between anions and cations, effectively preventing the decomposition of ionogel at high voltages [42]. Hence, it can be deduced that the strongest electrostatic interaction exists at a concentration of 31 vol%, resulting in the widest electrochemical stability window.

Cyclic Voltammetry (CV) is an electrochemical characterization technique employed on 2 electrodes, or 3-electrode configurations, to characterize liquid electrolyte-based capacitors. This technique provides information on charge storage at the individual interfaces [43]. In this study, CV was applied to characterize the specific capacitance of the synthesized ionogels, which depends on the accessibility of the surface area and ion concentration in the ionogel.

**Figure 7:**
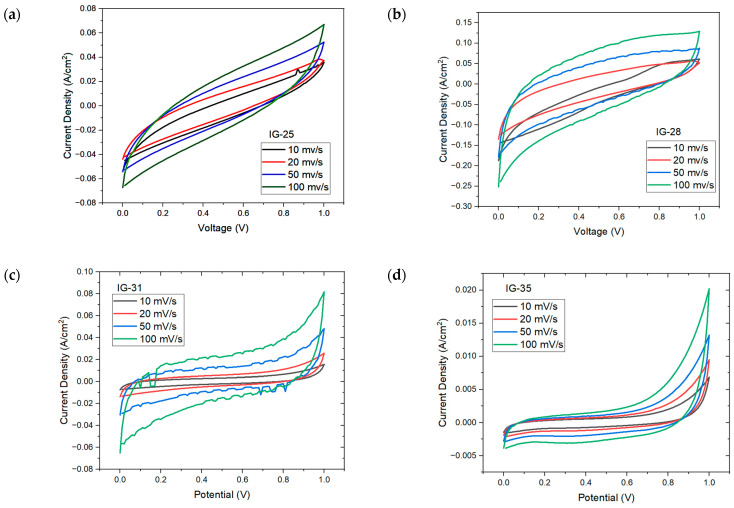

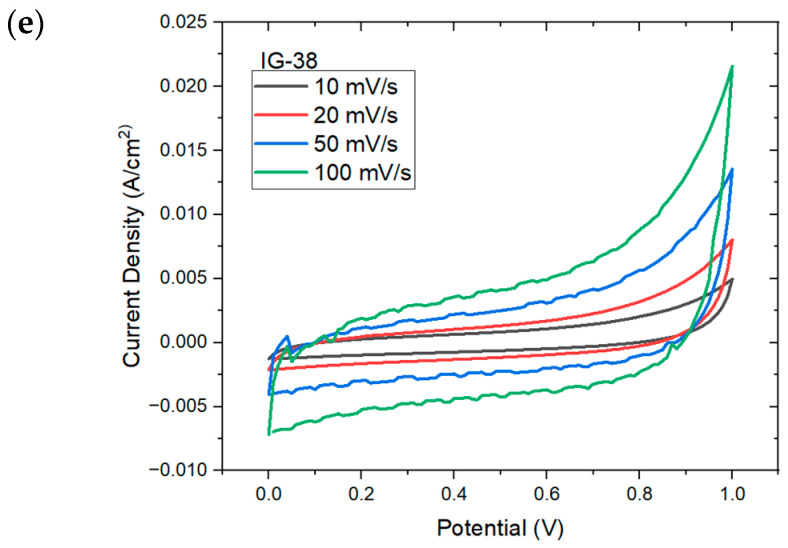
CV plots for (**a**) IG-25, (**b**) IG-28, (**c**) IG-31, (**d**) IG-35 (**e**) IG-38.

Figure 7 shows the CV plot for IG with varying concentration of electrolyte solution. At low concentration of electrolyte solution (less than 31 vol%), the CV plot displays an oval, ellipse-like shape, especially IG-25; this indicates the high instability and poor capacitive nature of ionogel, which is well reflected on the specific capacitance of IG-25 [44]. However, other than IG-25 and IG-28, all other figures show similar behavior, whereby a quasi-rectangular plot is observed; this implies excellent capacitive behavior and rapid charge propagation [45,46]. As the concentration of the electrolyte solution gradually increases, it can be observed that the CV plots approach rectangular shapes, up to 37 vol%. The presence of a rectangular CV plot implies pure double-layer capacitance. However, beyond this concentration, the CV plot drifts away from a rectangular shape. It was claimed that this results from the aggregation of ionic liquid. Meanwhile, the distorted CV plots could be due to the increasing internal resistance [47], Fontaine and Janani suggest that the deformed CV plots imply the resistive behavior of ionogel, behaving more like a resistor than a supercapacitor [48,49]. Moreover, the electrolyte–electrode charge transfer would also result in deviation from rectangular shape, while the reactions are considered pseudocapacitive provided that the charge transfer is reversible [50]. Furthermore, the increasing scan rates also increase the maximum current, which is one of the characteristics of capacitive material. In addition, no distinct peaks were observed in the CV plots due to internal resistance, implying a non-faradic process and charge double-layer at the electrode surface [51,52].

The cyclic voltammetry plot is also used to evaluate the specific capacitance of ionogel. The specific capacitance reflects charge storage at the interfacial region due to the interaction between the ions (from the electrolyte) and electrons (in the electrode) under an applied electric field [53]. The specific capacitance of ionogel is obtained at each scan rate and tabulated in Table 3.

Table 3 shows the highest specific capacitance value at 10 mV/s, where the specific capacitance ranges from 838.55 mF/g to 45.74 F/g, respectively. It is worth mentioning that the finding in specific capacitance is higher than the particular polymer capacitance electrolytes reported in the literature, which range only from about 1.7 F/g to 2.1 F/g in several studies [54,55,56]. The study reveals a decrease in specific capacitance with an increasing scan rate, possibly due to the charge per unit of time. Efficient ion conduction and double-layer formation at low scan rates lead to higher specific capacitance values [41]. At a high scan rate, there is insufficient time for the diffusion of ions to the surface of polymers, thus reducing specific capacitance [57,58].

To further elaborate, Specific capacitance is related to the storage capacity of the ionogel, indicating the number of free ions present in the ionogel [59]. At lower concentrations of electrolyte solution (below 31 vol%), there is a lack of charge-carrying ions for desorption and adsorption, thus resulting in low specific capacitance. However, at 31 vol% of the electrolyte solution, there is an adequate amount of ions present in the ionogel, and the surface area of the electrode can be fully exploited. This increased ion availability facilitates faster ionic responses. As a result, more ions diffuse and accumulate at the electrode–electrolyte interface, promoting double-layer formation and improving the ionogel’s specific capacitance [60].

**Figure 8:**
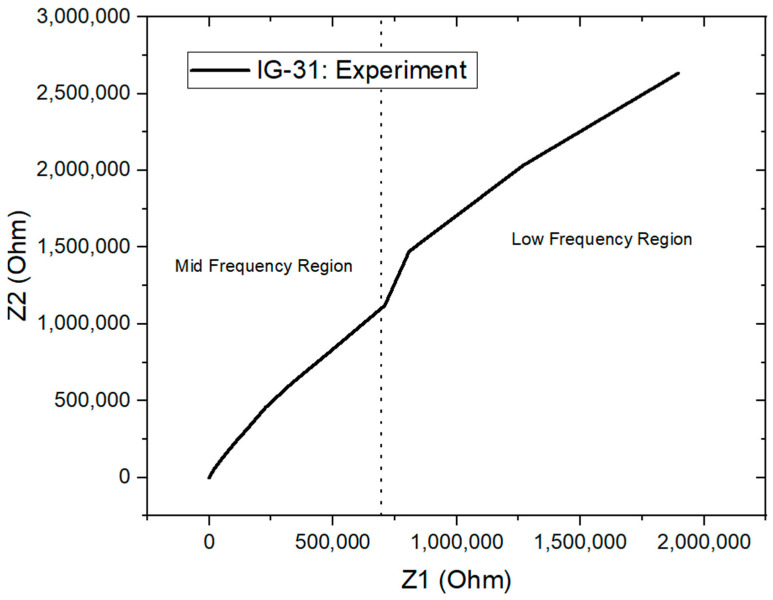
Nyquist plot for IG-31.

Electrochemical impedance spectroscopy (EIS) is performed on all the ionogel samples. This technique is considered definitive and effective in providing electrochemical characteristics of electrochemical systems. For instance, it provides information about solution resistance, double-layer capacitance, charge transfer process and charge transfer rate [61]. EIS is analyzed in a wide frequency range (0.01 Hz to 10 kHz) while the real and imaginary impedance is recorded, thus producing a Nyquist plot. The Nyquist plot can be differentiated into three regions according to their frequencies: the low-frequency region (f<0.1 Hz), mid-frequency region (1000 Hz<f<0.1 Hz) and ohmic region (f>1000 Hz). The Nyquist plot for IG-31 is shown in Figure 8 with frequency region labelled.

The ohmic region indicates the internal resistance of the ionogel; this is the region where the impedance of the ionogel switches from inductive behavior to capacitive behavior. Moreover, the mid-frequency region from the Nyquist plot is of interest in this study to study the ionic conductivity of ionogel. The ionic conductivity of the ionogel is evaluated with the equation below:

(1) $$\begin{array}{c} ionicc\;onductivity,\sigma =\frac{1}{R_{b}}\times \frac{L}{A} \end{array}$$

The mid-frequency region shows the charge transfer process from the surface of the electrode to the bulk active material of the electrodes and from the electrolyte to the electrode’s surface area [62]. Relating this to the equation for ionic conductivity, the $R_{b}$ refers to the bulk resistance, which is determined from this region. The equivalent circuit model fits the Nyquist plot to obtain bulk resistance.

**Scheme 1:**
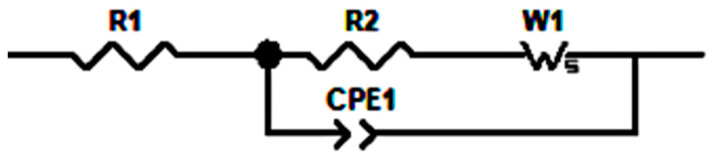
Equivalent Circuit Model.

The equivalent circuit model (ECM) is the common approach to evaluating the Nyquist plot by fitting the plot. The commonly used elements in an equivalent circuit model are resistor, constant phase, and Warburg element. A constant phase element (CPE) relates to the dispersion effect and how it differs from capacitance. The Warburg element (W) is used to interpret the mechanism of the diffusion process in the low-frequency region. The Nyquist plot from IG-31 to IG-38 shows a similar trend. Thus, the equivalent circuits are identical, as shown in Scheme 1, while the fitted Nyquist plot for IG-31is displayed in Figure 8. In the high-frequency region of the Nyquist plot, the inductive effects are observed and often show non-ideal behavior [63,64]. Thus, a resistor (R1) describes this phenomenon. The arc represents the impedance due to charge transfer for the mid-frequency region. In this region, these attributes are due to the combination of impedances, which can be modelled by the parallel combination of a resistor (R2) and a constant phase element (CPE1) [65]. Hence, the parallel combination of resistor and CPE represents the mid-frequency range, while the Warburg element fits the diffusion processes in the low-frequency region. In this study, the Warburg element is placed in series to R2 but parallel to CPE1; this corresponds to the consideration of simple geometry. This arrangement is also analyzed by Huang et al. [66], where from a physical perspective, the Warburg element connects to R2 in series while being parallel to CPE1 is preferred. The diffusion process followed by the charge transfer reaction is considered parallel to the discharging and charging process of the double layer [65].

**Figure 9:**
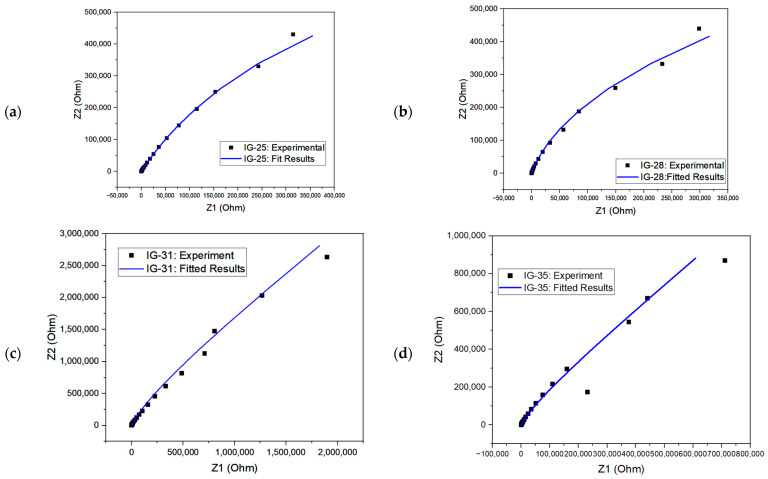

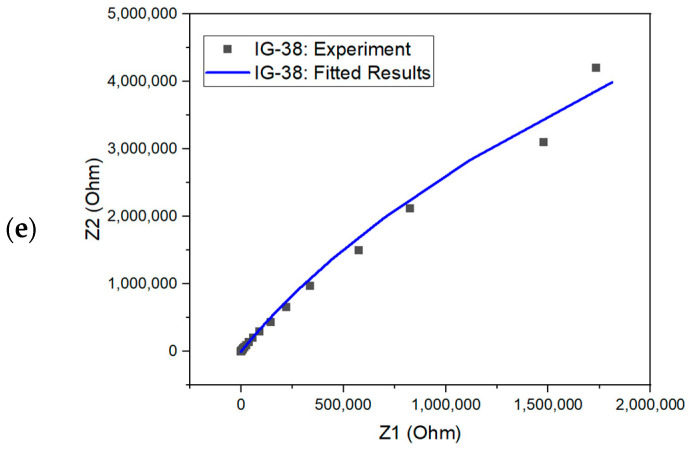
Nyquist plot with fitted and experiment results for (**a**) IG-25, (**b**) IG-28, (**c**) IG-31, (**d**) IG-35 and (**e**) IG-38.

From the equivalent circuit, the second resistor element (R2) refers to bulk resistance; hence, this value can be used to evaluate the ionic conductivity of ionogel [67,68]. The Nyquist plot for fitted and experimental results is shown in Figure 9. The simulated results could fit almost all the Nyquist plots. One way to examine the accuracy of the fitted results is through the chi-square value. The chi-square value indicates the discrepancy between the modelled data and the measured value of impedance analysis, whereby a larger chi-square value implies a larger deviation degree [69]. The chi-square value of each fitted model is within the range $10^{-3}–10^{-4}$, showing good fitting results.

Ionic conductivity is one of the crucial parameters for ionogel, while high ionic conductivity permits a good performance rate at a high charge/discharge density. The ionic conductivities are also tabulated in Table 4. The ionic conductivity of ionogel exhibits consistent behavior as in specific capacitance and electrochemical stability, where the maximum ionic conductivity is achieved by IG-31, followed by a substantial decrease. This finding is consistent with SEM and XRD results. Based on the SEM image, good compatibility between ionic liquid and the electrolyte solution is shown for IG-31, showing smooth and porous morphology, which enables ion transport, thus attaining high ionic conductivity.

Meanwhile, SEM results indicate that as the concentration of electrolyte solution increases, the porous morphology slowly disappears. As a result, ionic conductivity decreases as ion transportation relies on porous morphology. From the XRD analysis, IG-25 has the highest degree of crystallinity (72.46%). The degree of crystallinity gradually decreases as the concentration of electrolyte solution reaches 31 vol% (49.02%). Further increment of electrolyte solution leads to amplified crystallinity, whereby at 38 vol% of electrolyte solution, the degree of crystallinity has reached 63.51%. This XRD result is in complete unanimity with the ionic conductivity analysis of the ionogel. IG-25, which shows the highest crystallinity, impedes the transportation of ions, resulting in the lowest ionic conductivity (0.23±0.26 μS/cm) of ionogel. IG-31 possesses the lowest degree of crystallinity, showing the highest ionic conductivity (58±1.48 μS/cm).

The ionic conductivity value achieved by IG-31 is comparable to the values reported by recent literature; for instance, Barbosa et al. reported ionic conductivity of $3.9\times 10^{-5}\;S/cm$, including zeolite as filler, which is slightly lower than the values observed in our work [70]. Notably, in our work, the ionogel synthesized with a similar composition has achieved comparable ionic conductivity without adding any filler. This could be attributed to incorporating lithium salt, which provides more mobile ions. The observed result correlates with the findings from FTIR spectra, where the peak intensity (739 cm^−1^) increases as the concentration of electrolyte solution increases. Such a result is also in agreement with the results from cyclic voltammetry. A sufficient number of ions are available within the ionogel, corroborated by smooth and porous morphology, which assists the transport of ions, resulting in the highest ionic conductivity. However, a further increase in the electrolyte solution is accompanied by decreasing ionic conductivity. This is due to the formation of ion pairs which hinder the transport of ions. This is proven by a new peak in the FTIR spectra at wavelengths of 742 cm^−1^ and 1054 cm^−1^, respectively. In this study, the electrolyte solution is obtained by the dissolution of LiTf in EMIM TFSI. Lithium salts in ionic liquid could aid in enhancing the ionic conductivity of ionogel. Weak interaction exists within EMIM TFSI, while upon the addition of LiTf, this promotes the formation of ion pairs between Li^+^ ions and TFSI^−^ when more lithium salt becomes available [71]. Thus, excessive Li^+^ ions will interact with TFSI^−^. This interaction results in the aggregation of Li^+^ ions and TFSI- ions, which is responsible for the increased viscosity, hence decreasing the ionic conductivity; such behavior is also consistent with previous findings in the literature [72,73,74].

## 3. Experimental Sections

### 3.1. Synthesis of Ionogel

**Scheme 2:**
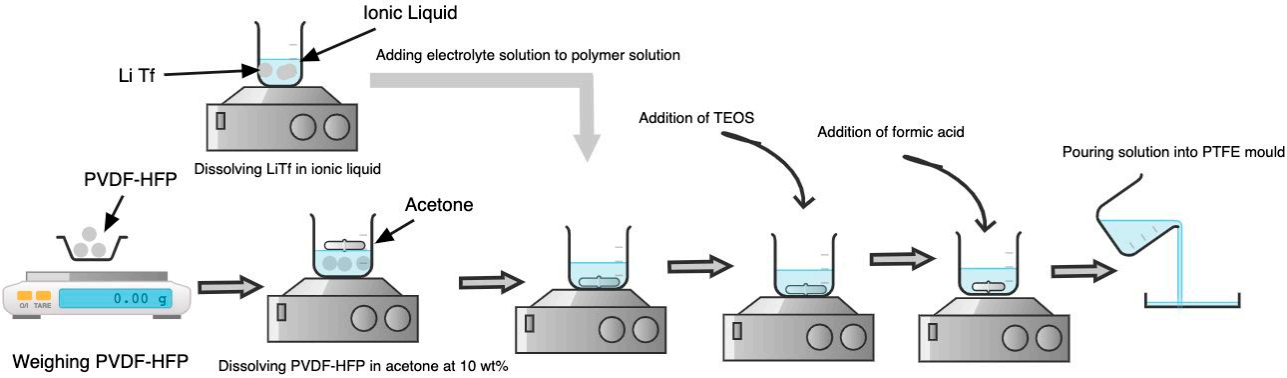
Synthesis of Ionogel.

The synthesis of ionogel was based on the non-aqueous sol-gel route. Scheme 2 illustrates the synthesis route for ionogel. The polymer matrix, PVDF-HFP, was first dissolved in acetone at 10 wt% for 24 h for complete dissolution. Beyond 10 wt%, the polymer could not be dissolved. The LiTf was then dissolved in ionic liquid to produce an electrolyte solution. After completely dissolving the polymer, the electrolyte solution was added to the polymer solution under continuous stirring conditions for another 1 h. After constant stirring for 1 h, the silica precursor, TEOS, was added to the solution for further mixing. The TEOS to polymer matrix ratio was maintained at 75:25. This ratio produces ionogel with good mechanical and electrical properties.

Meanwhile, the formic acid was added to the solution as a catalyst. The molar ratio of formic acid to TEOS was maintained at 7.8:1. After adding formic acid, the solution was cast onto the mold and left in an oven at room temperature for 2 days.

### 3.2. Material Characterization

The surface morphology of ionogel is characterized using SEM. X-ray diffraction (XRD) patterns were determined from a Rigaku Single X-ray Diffractometer (RGS, Selangor, Malaysia). The FTIR characterization was performed using Agilent Cary 630N (DKSH, Sarawak, Malaysia). The electrochemical characterization of ionogel was performed by an IVIUM compact state 10800 (Palico Biotech, Singapore). XRD was performed for 2θ between 5 to 80° at a scan rate of 1°/min. The crystallinity of ionogel was examined through this analysis, and the degree of crystallinity was determined based on the equation below:

(2) $$\begin{array}{c} Degree\;of\;crystallinity,X_{c}=\frac{A_{c}}{A_{c}\;+\;A_{a}}\times 100\% \end{array}$$

where $A_{c}$ and $A_{a}$ represent the area under the crystalline peak and the amorphous phase, respectively. The FTIR spectra were recorded in the wavelength of 600 cm^−1^ to 4000 cm^−1^ to inspect the conformational and structural changes of ionogel. The linear sweep voltammetry was performed in the range 0–5 V, with a 100 mV/s scan rate, to obtain the electrochemical stability of ionogel. The cyclic voltammetry was performed with different scan rates (10 mV/s, 20 mV/s, 50 mV/s and 100 mV/s), from 0 V to 1 V, while the specific capacitance of ionogel was determined based on the equation below:

(3) $$\begin{array}{c} C^{\prime }=\frac{\int Idv}{m\vartheta V} \end{array}$$

The integration was performed to obtain the area under the cure, while m is the mass of the ionogel (g), ϑ represents the scan rate (mV/s) and V is the voltage range. Electrochemical impedance spectroscopy was performed with the frequency range $0.01–10^{5}\;Hz$ to obtain impedance spectra. An equivalent circuit model was performed using the ivium software to identify the ionic conductivity of ionogel. An equivalent circuit model was performed using the ivium software to identify the ionic conductivity of ionogel. Thus, the equation below is used to evaluate ionic conductivity.

(4) $$\begin{array}{c} \sigma =\frac{1}{R}\frac{L}{S} \end{array}$$

where *σ*, *L* and *S* are the conductivity (S cm^−1^), thickness (cm) and electrode area (cm^2^), respectively.

## 4. Conclusions

In conclusion, this study has demonstrated a correlation between the specific capacitance and ionic conductivity of ionogels, which is dependent on the availability of mobile ions. Low ionic conductivity is achieved at lower concentrations of electrolyte solution due to limited mobile ions. Conversely, a higher concentration of electrolyte solution leads to the formation of ion pairs which reduces the ionic conductivity of ionogel. This interaction hinders ion mobility and results in the formation of double-layer capacitance, ultimately leading to diminished specific capacitance and ionic conductivity. In this study, 31 vol% is identified as the effective concentration, as an ample quantity of ions becomes accessible, resulting in increased ionic conductivity and specific capacitance with maximum values of 58 ± 1.48 μS/cm and 45.74 F/g, respectively. This finding is consistent with the outcomes derived from FTIR spectra, which exhibit an intensified intensity at 739 cm^−1^. The ionogel also demonstrates a wide electrochemical stability of 3.5 V and attains electrochemical properties suitable for diverse practical applications.

Consequently, this study offers critical insights into determining the optimal electrolyte solution concentration for enhancing ionogel electrochemical performance. It highlights the impact of ion pairs and aggregates on ion mobility within ionogels, subsequently affecting their electrochemical properties. This is the first study to report such outstanding ionic conductivity and electrochemical stability at a relatively low electrolyte concentration of around 30% without incorporating any filler material. This groundbreaking finding highlights the potential of ionogels in various applications while presenting new avenues for future research in the field.

## Figures and Tables

**Table 1:** Degree of Crystallinity for different concentrations of Electrolyte Solution.

Ionogel	Degree of Crystallinity (%)
IG-25	72.46
IG-28	52.16
IG-31	49.02
IG-35	55.66
IG-38	63.51

**Table 2:** Electrochemical stability of Ionogel with different compositions of electrolyte solution.

Ionogel-x vol% of Electrolyte Solution	Electrochemical Stability (V)
IG-25	3.00 ± 0.45
IG-28	3.20 ± 0.06
IG-31	3.50 ± 0.05
IG-35	3.20 ± 0.35
IG-38	3.10 ± 0.07

**Table 3:** Specific Capacitance of ionogel with different concentrations of electrolyte solution at different scan rates.

	10 mV/s	20 mV/s	50 mV/s	100 mV/s
IG-25	3.16 F/g	1.84 F/g	1.40 F/g	1.29 F/g
IG-28	28.73 F/g	11.55 F/g	7.63 F/g	6.23 F/g
IG-31	45.74 F/g	28.34 F/g	23.99 F/g	19.39 F/g
IG-35	10.75 F/g	7.93 F/g	4.53 F/g	3.89 F/g
IG-38	838.55 mF/g	719.832 mF/g	569.832 mF/g	470.949 mF/g

**Table 4:** Ionic Conductivity of Ionogels.

Ionogel	Ionic Conductivity (μS/cm)
IG-25	1.64±2.78
IG-28	2.74±1.27
IG-31	58.00±1.48
IG-33	1.77±4.22
IG-35	1.07±3.40
IG-37	0.14±0.33
IG-38	0.27±0.16

## Data Availability

Not applicable.

## Abbreviations

CV	Cyclic Voltammetry
EIS	Electrochemical Impedance Spectroscopy
EMIM TFSI	1-ethyl-3-methylimidazolium bis (trifluoromethyl sulfonate)
FTIR	Fourier transform infrared spectroscopy.
IG	Ionogel
IL	Ionic Liquid
LiTf	Lithium trifluoro methane sulfonate
LSV	Linear Sweep Voltammetry
PVDF-HFP	Poly(vinylidene fluoride)-co-hexafluoropropylene
SEM	Scanning Electron Microscopy
TEOS	Tetraethyl orthosilicate
XRD	X-ray diffraction
